# Perspectives and experiences regarding pre-exposure prophylaxis (PrEP) in a community sample of Veterans with unhealthy alcohol use: overall and across sexual orientation and gender identity

**DOI:** 10.1186/s13722-024-00533-y

**Published:** 2025-01-28

**Authors:** Olivia V. Fletcher, Kristine Beaver, Elizabeth J. Austin, Jenna van Draanen, E. Jennifer Edelman, Emily C. Williams

**Affiliations:** 1 Health Services Research & Development (HSR&D) Center of Innovation for Veteran-Centered and Value-Driven Care, Veterans Affairs (VA) Puget Sound Health Care System, 1660 S. Columbian Way, Mail Stop S-152, Seattle, WA 98108 USA; 2 Department of Health Systems and Population Health, University of Washington School of Public Health, Seattle, WA 98195 USA; 3 Yale School of Medicine, Center for Interdisciplinary Research on AIDS, Yale School of Public Health, New Haven, CT 06510 USA

**Keywords:** HIV, Pre-exposure prophylaxis, PrEP, Alcohol use, AUD, LGBTQ, Sexual and gender minorities

## Abstract

**Background:**

Unhealthy alcohol use is an independent, modifiable risk factor for HIV, but limited research addresses alcohol use and HIV prevention synergistically. Groups that experience chronic stigma, discrimination, and/or other marginalization, such as sexual and gender minoritized groups, may have enhanced HIV risk related to unhealthy alcohol use. We described awareness of and experiences with pre-exposure prophylaxis (PrEP) among a community sample of Veterans reporting unhealthy alcohol use (relative to those without), overall and across self-reported sexual orientation and gender identity.

**Methods:**

Using data collected from a national online survey of Veterans recruited via Facebook ads, community organizations, and listservs, we assessed variation in four PrEP outcomes (knowledge, experience, willingness, and conversations with doctors) across patterns of unhealthy alcohol use among all respondents reporting any lifetime drinking (*n* = 1,041) and then within sexual orientation and gender identity groups using Chi-square or Fisher’s exact tests.

**Results:**

Among 1,041 eligible Veterans, 440 (42%) screened positive for unhealthy alcohol use. Veterans with unhealthy alcohol use were not more likely to have heard of PrEP (58.2% vs. 55.4%, *p* = 0.37), but trended toward more likely to have taken PrEP (7.5% vs. 5.0%, *p* = 0.09), to be willing to take PrEP (30.5% vs. 27.6%, *p* = 0.06), and to have spoken with a doctor about PrEP (11.4% vs. 7.7%, *p* = 0.04). Those with heavy episodic drinking also trended toward higher prevalence of PrEP awareness (60.0% vs. 54.6%, *p* = 0.09), and were more likely to have taken PrEP (8.3% vs. 4.7%, *p* = 0.02), to be willing to use PrEP (34.6% vs. 25.5%, *p* < 0.01), and to have had conversations with doctors about PrEP (12.7% vs. 7.2%, *p* < 0.01). Similar patterns were observed for severe unhealthy alcohol use and past-year frequent heavy episodic drinking. Generally, sexual/gender minoritized Veterans with unhealthy alcohol use reported more PrEP-affirming responses than those without but associations with unhealthy alcohol use were similar.

**Conclusions:**

Unhealthy alcohol use was prevalent, particularly among Veterans with minoritized sexual orientation/gender identity, but not clearly linked to increased PrEP-literacy and use despite its known status as an HIV risk factor. Across groups, > 25% of individuals were willing to take PrEP. Interventions targeting both alcohol use and HIV prevention should capitalize on this.

## Background

In the United States, 1.7 million adults have HIV, which continues to be diagnosed at high rates: 37,981 diagnoses were made in the U.S. in 2022 [1]. Unhealthy alcohol use [2] is a common, important, and modifiable contributor to HIV incidence, which increases HIV risk via interference with decision making (re: safer sex and drug use practices) [3], lower benefit from HIV preventive interventions [4, 5], and poorer viral control among patients with HIV [6–9]. Though pre-exposure prophylaxis (PrEP)—antiretroviral medication now available both in pill and long-acting injection form—is highly effective at preventing HIV infection [10–12] and available, it continues to be underused. Preventing HIV transmission with enhanced PrEP uptake is a crucial step in ending the HIV epidemic [12–14], as even small increases in PrEP utilization could substantially decrease HIV incidence.

Despite the increased risk of HIV associated with alcohol use and the Centers for Disease Control and Prevention’s recommendation to screen for alcohol use as a potential indicator for PrEP [15], limited attention has been paid to PrEP among persons with unhealthy alcohol use [16–19]. Prior studies that have assessed PrEP awareness and experiences among persons with unhealthy alcohol use have often been conducted outside of the U.S., commonly in small samples of specific subpopulations, and largely have not been specifically focused on unhealthy alcohol use [18, 20–28]. Most alcohol-focused HIV prevention work has focused on treatment as prevention or improving viral control [16]. Studies are needed to understand PrEP awareness and experiences among persons with unhealthy alcohol use.

While unhealthy alcohol use is a risk factor for HIV in all groups, both unhealthy alcohol use and HIV disproportionately occur in marginalized populations, including persons with minoritized sexual and gender identities [29–36]. These groups experience chronic stigma, discrimination, and marginalization in a hetero- and cis-normative society, and experience multiple individual-level sequelae of these experiences (e.g., chronic stress and lack of healthcare access). Some of these factors (e.g., stigma, medical mistrust) may influence both unhealthy alcohol use and PrEP awareness [37–40]. Moreover, given increased unhealthy alcohol use and greater HIV prevalence in these groups, they may be key target populations for PrEP [13, 41–46]. Research combining a population-level approach and a vulnerable populations approach is needed to examine PrEP awareness and experiences in persons at greater risk due to their unhealthy alcohol use broadly and also across various sexual and gender identities [16, 29, 41–44].

U.S. Veterans are an important population in which to study these questions [47, 48]. Both persons with minoritized sexual and gender identities as well as persons reporting unhealthy alcohol use are disproportionately represented among U.S. Veterans [49], and recent research has shown that HIV prevalence in Veterans is highest in those with alcohol or opioid use disorders [50]. Moreover, the Veterans Health Administration (VA) is the nation’s largest provider of HIV care and offers multiple clinician resources for PrEP [48]. The descriptive analyses reported here address an important issue in HIV prevention—namely to what extent Veterans with unhealthy alcohol use are aware of and willing to take PrEP. Understanding this awareness/willingness and these experiences with PrEP among U.S. Veterans with unhealthy alcohol use and those at the intersection of unhealthy alcohol use and minoritized sexual and gender identity can help target resources for PrEP for Veterans. Therefore, in a national community sample of Veterans who responded to a survey, we assessed awareness of PrEP, willingness to take PrEP, history of taking PrEP, and whether or not their healthcare clinician had addressed PrEP as a preventive measure during their care for those with and without unhealthy alcohol use. There is no hypothesis due to the observational and exploratory nature of this study.

## Methods

### Data sources and population

This project represents a secondary analysis of data collected as part of a study designed to understand disparities in mental health and health risk behaviors among U.S. Veterans across sexual orientation and gender identity [51]. Study participants were Veterans who were recruited from September 2019 to December 2020, with a goal of recruiting 200 Veterans who were diverse based on sex, gender identity, and sexual orientation. The recruitment process was designed to enroll a community sample (not specific to those receiving care in the VA). Recruitment enlisted three approaches: (1) online ads distributed by 308 organizations (e.g., Veteran groups, LGBTQ Veteran groups, and the wider LGBTQ community); (2) Facebook ads; and (3) Social media ads via Trialfacts recruitment services. In the ads, the project was called the “Health for Every Veteran Study” and numerous ad headlines were used (e.g., “Online survey study for LGBT Veterans”, “LGBT Veteran health online research study”, and “Online study focusing on the health of every Veteran”). The study was further characterized with one of several descriptions of the research goals (e.g., “Researchers are focusing on understanding how Veterans’ identities, stressors, and experiences may affect their day-to-day life over time, including their mental, physical, and social health” and “This study wants to find out if the identities and life experience of Veterans with LGBT or related identities affect their health over time”). Interested Veterans were directed to an online information statement, consent, and screening process and were eligible if they were 18 years or older, had prior service in the military, U.S. residence, reliable internet access, valid contact information, and willingness to answer demographic questions. Veteran status was further verified with additional questions regarding military branch, job field and acronym, and rank. Exclusion criteria included providing nonsensical data and being presently incarcerated (per VA Institutional Review Board (IRB) regulations). Eligible participants were emailed directions for completing a 60–90 min online survey and were paid $30. Precautions were taken to limit misrepresentation and to ensure data validity (e.g., not advertising compensation amount, screening for Veteran status with “insider knowledge” questions and excluding surveys with illogical entries or completion time < 10 min). All study procedures were approved by the VA Puget Sound Healthcare System IRB.

### Measures

#### Demographics

Demographic measures included age, race, and ethnicity (Hispanic, non-Hispanic white, non-Hispanic Black, non-Hispanic other or multiracial), employment (full time, part time, retired, student, disabled or other, unemployed), marital status (married, never married, separated, divorced, widowed, other), and region of the U.S. based on state of residence (Northeast, South, Midwest, West).

#### Sex, gender identity, and sexual orientation

Sex and gender identity were determined via self-report of sex assigned at birth and current self-identified gender identity. Groups included cisgender, transgender, or another gender identity (nonbinary, genderqueer, gender fluid, or other), and participants indicated their sexual orientation as heterosexual, gay or lesbian, or bisexual.

Self-reported sex, gender identity, and sexual orientation were combined to group participants into the following nine subgroups: cisgender heterosexual men, cisgender gay men, cisgender bisexual men, transgender men, cisgender heterosexual women, cisgender lesbian women, cisgender bisexual women, transgender women, and other. Those whose survey responses indicated that they were transgender were not further split into sexual orientation groups.

#### Dependent variables of interest: patterns of unhealthy alcohol use

Participants completed the validated 3-item Alcohol Use Disorder Identification Test Consumption (AUDIT-C) questionnaire. AUDIT-C scores range from 0 to 12 [52–54] and higher scores indicate increased likelihood of alcohol use disorder (AUD) [52–55]. Veterans were considered to have past-year unhealthy alcohol use if they had an AUDIT-C score *≥* 3 drinks per day for cisgender women, transgender women, and other gender or *≥* 4 drinks per day for cisgender men and transgender men on a typical drinking day. Severe unhealthy alcohol use was indicated by an AUDIT-C score of *≥* 8 regardless of gender, consistent with prior research [56]. Heavy episodic drinking was indicated by any response other than “never” to the third AUDIT-C question “How often in the last year have you had six or more drinks on one occasion?”, and frequent heavy episodic drinking was indicated by a response of “monthly”, “weekly”, or “daily”.

#### Pre-exposure prophylaxis (PrEP) outcomes of interest

Participants answered four questions regarding PrEP awareness/use: (1) Have you ever heard of pre-exposure prophylaxis (PrEP) to prevent HIV infection?, (2) Have you ever taken PrEP to prevent HIV infection?, (3) Would you be willing to use PrEP if it was available?, (4) Has your doctor ever talked with you about using PrEP to prevent HIV infection? Willingness to take PrEP was the only non-binary question of the four; answer choices for this question were yes, no, and I don’t know.

### Statistical analysis

We described measures of unhealthy alcohol use as well as PrEP-affirming responses, overall and across sexual orientation and gender identity subgroups. Then, within the total sample and stratified by sexual orientation and gender identity, we assessed variation in the PrEP-affirming response rate across each measure of unhealthy alcohol use. We used Chi-Square or Fisher’s exact test to compare groups depending on expected values and used a *p*-value of < 0.05 as a cutoff for statistical significance. Analyses were conducted using Stata/SE, version 16.0.

## Results

Among 1,041 eligible Veterans, the majority reported non-Hispanic White race and ethnicity (78.6%) and were well-distributed across age categories (except for those 25 and under (2.4%)) (Table 1). Most participants identified as a cisgender man or woman (39.8% and 39.0%, respectively), though 10.% identified as transgender women. Further, 37.8% self-identified as lesbian or gay, 32.9% as straight or heterosexual, 14.5% as bisexual, and 14.9% as other.

**Table 1:** Characteristics of *N* = 1,041 veteran survey respondents

	*N* (%)
Female sex at birth	462 (44.5)
Age (years) (mean, SD)	51.1 (14.8)
Age categories	
<=25	25 (2.4)
26–35	170 (16.3)
36–45	210 (20.2)
46–55	206 (19.8)
56–65	221 (21.2)
>65	209 (20.1)
Race/ethnicity	
Hispanic	89 (8.6)
White (NH)	818 (78.6)
Black (NH)	48 (4.6)
Other or multiracial (NH)	84 (8.1)
Employment status	
Full-time paid	414 (39.9)
Part-time paid	118 (11.4)
Retired	289 (27.8)
Student	45 (4.3)
Disabled	75 (7.2)
Unemployed	69 (6.6)
Other	29 (2.8)
Education	
High school diploma or GED	46 (4.4)
Some college/trade school/associate’s	395 (38.0)
College graduate	225 (21.6)
Some graduate/professional school	85 (8.2)
Postgraduate degree	289 (27.8)
Marital status	
Married or domestic partnership	535 (51.4)
Never married	198 (19.0)
Separated	33 (3.2)
Divorced	225 (21.6)
Widowed	33 (3.2)
Other	16 (1.5)
Region of residence	
Northeast	114 (11.0)
South	428 (41.2)
Midwest	205 (19.7)
West	293 (28.2)
Current gender identity	
Man	414 (39.8)
Woman	406 (39.0)
Transgender man	36 (3.5)
Transgender woman	107 (10.3)
Genderqueer	67 (6.4)
Other	10 (1.0)
Sexual orientation	
Lesbian or gay	393 (37.8)
Straight or heterosexual	342 (32.9)
Bisexual	151 (14.5)
Other	155 (14.9)
AUDIT-C score (mean, SD)	3.0 (2.7)
Unhealthy alcohol use	440 (42.3)
Severe unhealthy alcohol use	84 (8.1)
Heavy episodic drinking	385 (37.0)
Frequent heavy episodic drinking	147 (14.1)

**Fig. 1:**
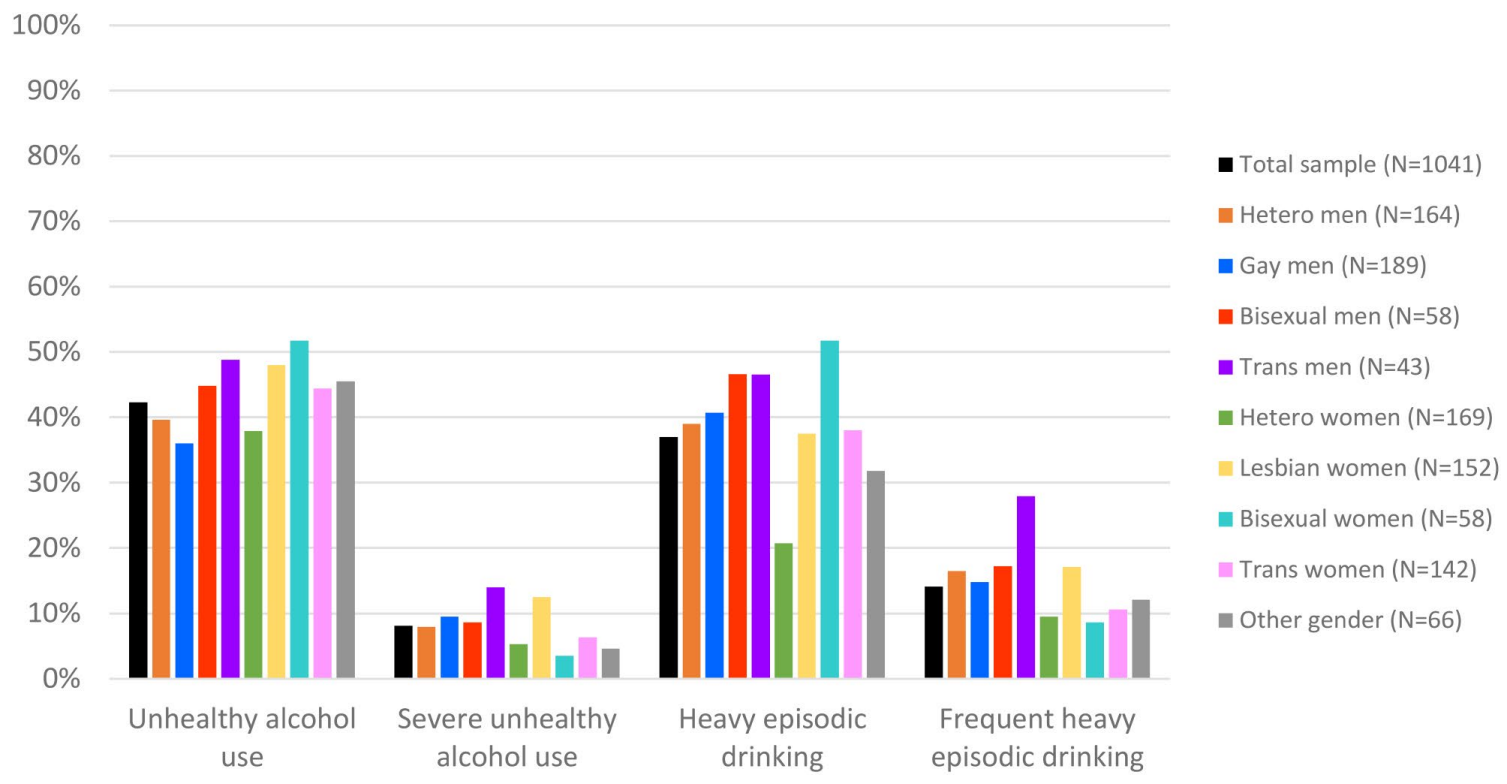
Patterns of unhealthy alcohol use in a community sample of Veterans across sexual and gender identity

The median AUDIT-C score among all participants was 2 (IQR 1–4) (Table 1). Among all participants, 440 Veterans (42.3%) screened positive for unhealthy alcohol use, 84 (8.1%) for severe unhealthy alcohol use, 385 (37.0%) for heavy episodic drinking, and 147 (14.1%) for frequent heavy episodic drinking (Fig. 1; Table 2). Prevalence of unhealthy alcohol use and heavy episodic drinking were generally higher in Veterans identifying as sexual/gender minorities, however, gay men reported the lowest prevalence of unhealthy alcohol use (36.0%). Transgender men had the highest prevalence of both severe unhealthy alcohol use (14.0%) and frequent heavy episodic drinking (27.9%). Bisexual women had the highest prevalence of heavy episodic drinking (51.7%), followed closely by bisexual men (46.6%) and transgender men (46.5%) (Fig. 1; Table 2).

 Please replace this Figure 1 with the newly uploaded Figure 1.

**Table 2:** N (%) of veteran survey respondents indicating patterns of unhealthy alcohol use

	Unhealthy alcohol use	Severe unhealthy alcohol use	Heavy episodic drinking	Frequent heavy episodic drinking
Total sample (*N* = 1041)	440 (42.3)	84 (8.1)	385 (37.0)	147 (14.1)
Hetero men (*N* = 164)	65 (39.6)	13 (7.9)	64 (39.0)	27 (16.5)
Gay men (*N* = 189)	68 (36.0)	18 (9.5)	77 (40.7)	28 (14.8)
Bisexual men (*N* = 58)	26 (44.8)	5 (8.6)	27 (46.6)	10 (17.2)
Trans men (*N* = 43)	21 (48.8)	6 (14.0)	20 (46.5)	12 (27.9)
Hetero women (*N* = 169)	64 (37.9)	9 (5.3)	35 (20.7)	16 (9.5)
Lesbian women (*N* = 152)	73 (48.0)	19 (12.5)	57 (37.5)	26 (17.1)
Bisexual women (*N* = 58)	30 (51.7)	2 (3.5)	30 (51.7)	5 (8.6)
Trans women (*N* = 142)	63 (44.4)	9 (6.3)	54 (38.0)	15 (10.6)
Other gender (*N* = 66)	30 (45.5)	3 (4.6)	21 (31.8)	8 (12.1)

### Results in the overall sample

Among 1,041 eligible Veterans, 440 (42%) screened positive for unhealthy alcohol use. Veterans with unhealthy alcohol use were not more likely to have heard of PrEP than those without unhealthy alcohol use (58.2% vs. 55.4%, *p* = 0.37), but trended toward being more likely to have taken PrEP (7.5% vs. 5.0%, *p* = 0.09), to be willing to take PrEP (30.5% vs. 27.6%, *p* = 0.06) and to have spoken with a doctor about PrEP (11.4% vs. 7.7%, *p* = 0.04) (Table 3). In the overall sample (not split into sexual and gender subgroups), only those reporting heavy episodic drinking trended toward being more likely to be aware of PrEP (60.0% vs. 54.6%, *p* = 0.09). However, those reporting any of the four patterns of unhealthy alcohol use trended toward greater likelihood of endorsing the other PrEP measures with the exception of those with severe unhealthy alcohol use and willingness to use PrEP (35.7% vs. 28.2%, *p* = 0.13). Significant findings in the total sample included: Veterans with heavy episodic drinking and frequent heavy episodic drinking were more likely to have taken PrEP (8.3% vs. 4.7%, *p* = 0.02 and 10.2% vs. 5.4%, *p* = 0.02, respectively), to be willing to take PrEP (34.6% vs. 25.5%, *p* < 0.01 and 36.1% vs. 27.6%, *p* = 0.04, respectively), and to have spoken with a doctor about PrEP (12.7% vs. 7.2%, *p* < 0.01 and 16.3% vs. 8.1%, *p* < 0.01, respectively). Veterans reporting unhealthy alcohol use and severe unhealthy alcohol use were also significantly more likely to have spoken with a doctor about PrEP (11.4% vs. 7.7%, *p* = 0.04 and 16.7% vs. 8.6%, *p* = 0.01, respectively).

**Table 3:** N (%) of veteran survey respondents answering affirmatively to questions regarding pre-exposure prophylaxis (PrEP) across four patterns of unhealthy alcohol use

		Overall		Unhealthy alcohol use			Severe unhealthy alcohol use			Heavy episodic drinking			Frequent heavy episodic drinking		
		N (%)		No	Yes	*p*-value	No	Yes	*p*-value	No	Yes	*p*-value	No	Yes	*p*-value
Aware of PrEP	Total sample (*N* = 1041)	588 (56.5)		333 (55.4)	256 (58.2)	0.37	540 (56.4)	49 (58.3)	0.74	358 (54.6)	231 (60.0)	0.09	503 (56.3)	86 (58.5)	0.61
	Hetero men (*N* = 164)	46 (28.1)		31 (31.3)	15 (23.1)	0.25	43 (28.5)	3 (23.1)	1.00*	28 (28.0)	18 (28.1)	0.99	41 (29.9)	5 (18.5)	0.23
	Gay men (*N* = 189)	160 (84.7)		98 (81.0)	62 (91.2)	0.06	143 (83.6)	17 (94.4)	0.32*	92 (82.1)	68 (88.3)	0.25	133 (82.6)	27 (96.4)	0.09*
	Bisexual men (*N* = 58)	48 (82.8)		26 (81.3)	22 (84.6)	1.00*	44 (83.0)	4 (80.0)	1.00*	25 (80.7)	23 (85.2)	0.74*	40 (83.3)	8 (80.0)	1.00*
	Trans men (*N* = 43)	27 (62.8)		12 (54.6)	15 (71.4)	0.25	22 (59.5)	5 (83.3)	0.39*	15 (65.2)	12 (60.0)	0.72	18 (58.1)	9 (75.0)	0.48*
	Hetero women (*N* = 169)	61 (36.1)		36 (34.3)	25 (39.1)	0.53	61 (38.1)	0 (0.00)	0.03*	47 (35.1)	14 (40.0)	0.59	58 (37.9)	3 (18.8)	0.13
	Lesbian women (*N* = 152)	81 (53.3)		42 (53.2)	39 (53.4)	0.97	71 (53.4)	10 (52.6)	0.95	49 (51.6)	32 (56.1)	0.59	66 (52.4)	15 (57.7)	0.62
	Bisexual women (*N* = 58)	36 (62.1)		14 (50.0)	22 (73.3)	0.07	34 (60.7)	2 (100.0)	0.52*	16 (57.1)	20 (66.7)	0.46	32 (60.4)	4 (80.0)	0.64*
	Trans women (*N* = 142)	90 (63.4)		52 (65.8)	38 (60.3)	0.50	85 (63.9)	5 (55.6)	0.72*	59 (67.1)	31 (57.4)	0.25	82 (64.6)	8 (53.3)	0.39
	Other gender (*N* = 66)	39 (60.0)		22 (61.1)	18 (60.0)	0.93	37 (58.7)	3 (100.0)	0.27*	27 (60.0)	13 (61.9)	0.88	33 (56.9)	7 (87.5)	0.13*
Has taken PrEP	Total sample (*N* = 1041)	63 (6.1)		30 (5.0)	33 (7.5)	0.09	54 (5.6)	9 (10.7)	0.06	31 (4.7)	32 (8.3)	0.02	48 (5.4)	15 (10.2)	0.02
	Hetero men (*N* = 164)	1 (0.6)		1 (1.0)	0 (0.0)	1.00*	1 (0.7)	0 (0.0)	1.00*	1 (1.0)	0 (0.0)	1.00*	1 (0.7)	0 (0.0)	1.00*
	Gay men (*N* = 189)	40 (21.2)		22 (18.2)	18 (26.5)	0.18	36 (21.1)	4 (22.2)	1.00*	19 (17.0)	21 (27.3)	0.09	31 (19.3)	9 (32.1)	0.12
	Bisexual men (*N* = 58)	5 (8.6)		1 (3.1)	4 (15.4)	0.16*	4 (7.6)	1 (20.0)	0.37*	2 (6.5)	3 (11.1)	0.66*	4 (8.3)	1 (10.0)	1.00*
	Trans men (*N* = 43)	3 (7.0)		1 (4.6)	2 (9.5)	0.61*	1 (2.7)	2 (33.3)	0.05*	1 (4.4)	2 (10.0)	0.59*	1 (3.2)	2 (16.7)	0.18*
	Hetero women (*N* = 169)	0 (0.0)		0 (0.0)	0 (0.0)	---	0 (0.0)	0 (0.0)	---	0 (0.0)	0 (0.0)	---	0 (0.0)	0 (0.0)	---
	Lesbian women (*N* = 152)	2 (1.3)		0 (0.0)	2 (2.7)	0.23*	1 (0.8)	1 (5.3)	0.24*	0 (0.0)	2 (3.5)	0.14*	1 (0.8)	1 (3.9)	0.31*
	Bisexual women (*N* = 58)	0 (0.0)		0 (0.0)	0 (0.0)	---	0 (0.0)	0 (0.0)	---	0 (0.0)	0 (0.0)	---	0 (0.0)	0 (0.0)	---
	Trans women (*N* = 142)	9 (6.3)		5 (6.3)	4 (6.4)	1.00*	8 (6.0)	1 (11.1)	0.46*	6 (6.8)	3 (5.6)	1.00*	7 (5.5)	2 (13.3)	0.24*
	Other gender (*N* = 66)	3 (4.6)		0 (0.0)	3 (10.0)	0.09*	3 (4.8)	0 (0.0)	1.00*	2 (4.4)	1 (4.8)	1.00*	3 (5.2)	0 (0.0)	1.00*
Willing to take PrEP	Total sample (*N* = 1041)	299 (28.8)	166 (27.6)		134 (30.5)	0.06	270 (28.2)	30 (35.7)	0.13	167 (25.5)	133 (34.6)	< 0.01	247 (27.6)	53 (36.1)	0.04
	Hetero men (*N* = 164)	22 (13.4)	12 (12.1)		10 (15.4)	0.38	21 (13.9)	1 (7.7)	0.12*	11 (11.0)	11 (17.2)	0.35	20 (14.6)	2 (7.4)	0.02*
	Gay men (*N* = 189)	96 (51.1)	55 (45.5)		41 (60.3)	0.03	86 (50.3)	10 (55.6)	0.80*	50 (44.6)	46 (59.7)	0.07	77 (47.8)	19 (67.9)	0.14
	Bisexual men (*N* = 58)	34 (58.6)	16 (50.0)		18 (69.2)	0.29*	29 (54.7)	5 (100.0)	0.25*	16 (51.6)	18 (66.7)	0.53*	25 (52.1)	9 (90.0)	0.11*
	Trans men (*N* = 43)	16 (37.2)	6 (27.3)		10 (47.6)	0.36	12 (32.4)	4 (66.7)	0.47*	7 (30.4)	9 (45.0)	0.57	8 (25.8)	8 (66.7)	0.07*
	Hetero women (*N* = 169)	14 (8.3)	11 (10.5)		3 (4.7)	0.36	13 (8.1)	1 (11.1)	0.18*	12 (9.0)	2 (5.7)	0.84*	13 (8.5)	1 (6.3)	0.72*
	Lesbian women (*N* = 152)	20 (13.2)	12 (15.2)		8 (11.0)	0.36	17 (12.8)	3 (15.8)	0.84*	12 (12.6)	8 (14.0)	0.94	15 (11.9)	5 (19.2)	0.58*
	Bisexual women (*N* = 58)	16 (27.6)	9 (32.1)		7 (23.3)	0.55	16 (28.6)	0 (0.0)	1.00*	9 (32.1)	7 (23.3)	0.75	15 (28.3)	1 (20.0)	0.86*
	Trans women (*N* = 143)	53 (37.3)	28 (35.4)		25 (39.7)	0.81	49 (36.8)	4 (44.4)	0.82*	30 (34.1)	23 (42.6)	0.48	48 (37.8)	5 (33.3)	0.47*
	Other gender (*N* = 66)	28 (43.1)	17 (47.2)		12 (40.0)	0.84	27 (42.9)	2 (66.6)	1.00*	20 (44.4)	9 (42.9)	0.83*	26 (44.8)	3 (37.5)	0.89*
Has spoken with provider about PrEP	Total sample (*N* = 1041)	95 (9.1)	46 (7.7)		50 (11.4)	0.04	82 (8.6)	14 (16.7)	0.01	47 (7.2)	49 (12.7)	< 0.01	72 (8.1)	24 (16.3)	< 0.01
	Hetero men (*N* = 164)	1 (0.6)	0 (0.0)		1 (1.5)	0.40*	1 (0.7)	0 (0.0)	1.00*	1 (1.0)	0 (0.0)	1.00*	1 (0.7)	0 (0.0)	1.00*
	Gay men (*N* = 189)	55 (29.1)	32 (26.5)		23 (33.8)	0.28	46 (26.9)	9 (50.0)	0.04	26 (23.2)	29 (37.7)	0.03	40 (24.8)	15 (53.6)	< 0.01
	Bisexual men (*N* = 58)	7 (12.1)	2 (6.3)		5 (19.2)	0.23*	5 (9.4)	2 (40.0)	0.11*	3 (9.7)	4 (14.8)	0.69*	5 (10.4)	2 (20.0)	0.59*
	Trans men (*N* = 43)	5 (11.6)	2 (9.1)		3 (14.3)	0.66*	4 (10.8)	1 (16.7)	0.55*	2 (8.7)	3 (15.0)	0.65*	3 (9.7)	2 (16.7)	0.61*
	Hetero women (*N* = 169)	2 (1.2)	2 (1.9)		0 (0.0)	0.53*	2 (1.3)	0 (0.0)	1.00*	2 (1.5)	0 (0.0)	1.00*	2 (1.3)	0 (0.0)	1.00*
	Lesbian women (*N* = 152)	4 (2.6)	1 (1.3)		3 (4.1)	0.35*	3 (2.3)	1 (5.3)	0.42*	1 (1.1)	3 (5.3)	0.15*	2 (1.6)	2 (7.7)	0.14*
	Bisexual women (*N* = 58)	1 (1.7)	0 (0.0)		1 (3.3)	1.00*	1 (1.8)	0 (0.0)	1.00*	0 (0.0)	1 (3.3)	1.00*	1 (1.9)	0 (0.0)	1.00*
	Trans women (*N* = 142)	15 (10.6)	5 (6.3)		10 (15.9)	0.07	14 (10.5)	1 (11.1)	1.00*	8 (9.1)	7 (13.0)	0.47	12 (9.5)	3 (20.0)	0.20*
	Other gender (*N* = 66)	5 (7.7)	2 (5.6)		4 (13.3)	0.40*	6 (9.5)	0 (0.0)	1.00*	4 (8.9)	2 (9.5)	1.00*	6 (10.3)	0 (0.0)	1.00*

* fisher exact test instead of chi2 used due to expected cell count < 5

### Results in heterosexual men and woman

Heterosexual women with severe unhealthy alcohol use were significantly less likely to be aware of PrEP than those without severe unhealthy alcohol use (0.0% vs. 38.1%, *p* = 0.03). Heterosexual men with frequent heavy episodic drinking were less likely to be willing to take PrEP than heterosexual men without (7.4% vs. 14.6%, *p* = 0.02) (Table 3). No other findings among heterosexual men and women were significant.

### Results in minoritized sexual and gender identity groups

Regarding awareness of PrEP among Veterans identifying as a minoritized sexual orientation or gender identity, few results trended toward significance. Gay men with unhealthy alcohol use and frequent heavy episodic drinking trended toward increased awareness of PrEP relative to those without unhealthy alcohol use (91.2% vs. 81.0%, *p* = 0.06 and 96.4% vs. 82.6%, *p* = 0.09, respectively) and bisexual women with unhealthy alcohol use trended toward increased awareness of PrEP (73.3% vs. 50.0%, *p* = 0.07) (Table 3). Gay men with heavy episodic drinking trended toward increased likelihood of taking PrEP (27.3% vs. 17.0%, *p* = 0.09) and transgender men with severe unhealthy alcohol use were more likely to have taken PrEP (33.3% vs. 2.7%, *p* = 0.05). Gay men with unhealthy alcohol use and heavy episodic drinking were more likely to be willing or trended toward greater willingness to take PrEP (60.3% vs. 45.5%, *p* = 0.03 and 59.7% vs. 44.6%, *p* = 0.07, respectively). Transgender men with frequent heavy episodic drinking also trended toward increased willingness to take PrEP (66.7% vs. 25.8%, *p* = 0.07). Gay men with severe unhealthy alcohol use, heavy episodic drinking, and frequent heavy episodic drinking were more likely to report having spoken to a doctor about PrEP (50.0% vs. 26.9%, *p* = 0.04; 37.7% vs. 23.2%, *p* = 0.03; and 53.6% vs. 24.8%, *p* < 0.01, respectively). Transgender women with unhealthy alcohol use also trended toward greater likelihood of having spoken with a doctor about PrEP (15.9% vs. 6.3%, *p* = 0.07) (Table 3).

All significant results among sexual and gender minoritized Veterans highlighted unhealthy alcohol use patterns associated with increased likelihood of endorsing PrEP measures. Statistically significant results regarding unhealthy alcohol use patterns and PrEP literacy among heterosexual Veterans in this study were scarce but when they did emerge, they were in the opposite direction than what was seen in sexual and gender minoritized Veterans—heterosexual Veterans with an unhealthy alcohol use pattern were less likely to endorse PrEP measures than those with an unhealthy alcohol use pattern (e.g., heterosexual women with severe unhealthy alcohol use re: awareness and heterosexual men with frequent heavy episodic drinking re: willingness).

Of note, no heterosexual women with severe unhealthy alcohol use reported having heard of PrEP and no heterosexual or bisexual women reported ever having taken PrEP (Table 3).

## Discussion

In this study of a community-based sample of U.S. Veterans, unhealthy alcohol use was extremely common. However, despite unhealthy alcohol use being a known and modifiable risk factor for HIV, experience taking PrEP and history of speaking with a doctor about PrEP among these patients were remarkably low (7.5% and 11.4%, respectively). Over half the total sample reported awareness of PrEP (range 55–60% across patterns of unhealthy alcohol use) and across patterns of unhealthy alcohol use, around a third reported willingness to take PrEP, which could provide a strong foundation for increasing PrEP utilization.

In the present study, though respondents with varying patterns of unhealthy alcohol use had higher proportions reporting having taken PrEP and talking with a doctor about PrEP than those without, differences were generally small. Because this study was solely descriptive, it is unclear whether differences observed are real or could be accounted for by other factors. It is possible that proportions of PrEP awareness and knowledge were slightly higher among persons with unhealthy alcohol use in this study due to increased HIV risk associated with unhealthy alcohol use. However, observed differences could also be due to higher rates of related chronic or acute medical conditions and thus more frequent utilization of healthcare, which could increase knowledge of and/or experience with PrEP. Further research in larger samples is needed to understand potential mechanisms and confirm findings, as differences were small and there were low proportions of persons endorsing awareness of and experiences with PrEP regardless of patterns of unhealthy alcohol use.

This information provides an important foundation for additional observational and intervention work to increase PrEP knowledge, willingness, and use among Veterans—particularly those with unhealthy alcohol use who are at increased risk of HIV incidence. Our findings may also support implementation work with clinicians and clinics to increase discussions of PrEP. Variation in PrEP awareness, history, willingness, and discussions with providers was observed overall and across subgroups based on sexual orientation and gender identity. Men of varying sexual orientations and transgender women appeared to have greater awareness of, history with, and openness to PrEP than women and transgender men across measures. Patterns of unhealthy alcohol use did not appear to have a tremendous correlation with PrEP-affirmative knowledge/experience across sexual and gender groups. However, though limited, the significant findings among individual sexual and gender groups corroborated a broader observed pattern: unhealthy alcohol use patterns appeared to often be associated with higher rates of PrEP-affirming responses among sexual and gender minorities but with lower rates of PrEP-affirming responses among heterosexual persons. These opposing trends observed between sexual and gender minorities and heterosexual persons could be explained in part by increased HIV- and PrEP-literacy among sexual and gender minority persons [29, 57–59], which may translate to heightened awareness of the increased risk of HIV associated with unhealthy alcohol use, while cisgender heterosexual persons remain less likely to consider the risk of HIV at all, let alone the compounded risk conveyed by alcohol use. Alternatively (or additionally), historic and/or dated perceptions of HIV risk could lead providers to be more likely to associate alcohol use with increased HIV risk only among sexual and gender minoritized persons and thus, more likely to broach the subject of PrEP with sexual and gender minoritized persons who use alcohol than with cisgender heterosexual persons who do. Future research is needed to understand patterns observed in the present study and to increase PrEP awareness across all groups. Because research has shown that unhealthy alcohol use occurs more frequently in sexual and gender minorities than in heterosexual persons [29, 31–34, 60, 61], that sexual and gender minorities are at greater risk of acquiring HIV [29, 35], and that alcohol consumption has been identified as a barrier to willingness to use PrEP [20], intervention work may be particularly important in these populations despite higher observed awareness of PrEP in these groups in the present study.

This study’s findings regarding women are noteworthy. Though women are broadly less likely than men to receive PrEP (they account for 19% of new HIV diagnoses but make up less than 5% of individuals taking PrEP [62, 63]), women in this sample were also largely unaware of PrEP. No heterosexual or bisexual women in our sample reported having ever taken PrEP. Additionally, zero heterosexual women with severe unhealthy alcohol use reported having heard of PrEP compared to 38.1% of heterosexual women without severe unhealthy alcohol use. Though it is possible that unknown confounders (e.g., reduced access to health care, reduced health-literacy, or reduced feelings of empowerment regarding health behaviors) are impacting both alcohol use and PrEP-literacy in the current study, present findings suggest a strong need for additional research on PrEP among women who use alcohol.

It is important to note that while this study conveys that doctors are not routinely talking to their patients about PrEP, in our sample of gay men, all patterns of alcohol use were significantly associated with the likelihood of having had these conversations with a doctor. While these findings were encouraging, a stark disparity was observed across sexual and gender subgroups regarding conversations with doctors about PrEP—no heterosexual women and only 7.7% of lesbian women with frequent heavy episodic drinking reported having talked with a doctor about PrEP compared to more than half (53.6%) of gay men with frequent heavy episodic drinking. Future work is needed to promote awareness among other sexual and gender groups who may be at risk but may also be more likely to be overlooked by clinicians based on the historical epidemiology of HIV.

These findings build upon those of prior studies regarding PrEP and alcohol use in samples of men who have sex with men (MSM) within [19, 64–66] and outside of [23, 67, 68] the VA. However, this is the first study to our knowledge to assess associations between unhealthy alcohol use patterns and PrEP knowledge/uptake across multiple marginalized subgroups, and while these data display encouraging results regarding increased PrEP literacy among Veterans with any pattern of unhealthy alcohol use, breaking these data up across sexual orientation and gender identity subgroups highlights that there are many subpopulations who are still not aware of or receiving PrEP despite engaging in a known HIV risk behavior—unhealthy alcohol use.

### Limitations

Our study has several limitations. First, we must acknowledge a critical limitation in our study population: only 4% of respondents were Black and 8% were Hispanic. Both of these populations are known to be at greater risk for HIV [51, 69], both groups have been shown to have PrEP literacy and prescribing rates that differ from white persons and/or the overall population [36, 70, 71], and both experience unhealthy alcohol use at levels differential to white persons and/or overall population [72–76]. For these reasons, our findings cannot be thought to represent the general population or subpopulations of Black and/or Hispanic people. Second, convenience sampling from online advertisements introduces potential response bias; respondents/participants may differ from persons who did not reply to the advertisements. Those who responded were people who could use a computer and/or phone/tablet and were also possibly persons with a greater interest in research or health, and as such, perhaps a greater awareness of PrEP or their HIV risk. Next, it is important to note that openness or willingness to take PrEP does not necessarily indicate intent to use PrEP (these have been identified as two distinct stages of the motivational PrEP cascade) [36], and further research should be done to explore what factors might act as barriers or facilitators to moving from willingness to intent, with a particular eye toward those who engage in substance use. Another limitation was that the sample was not equally distributed across sexual/gender groups, and unhealthy alcohol use patterns were not equally distributed within groups, which may have limited our ability to compare groups and to observe trends that might otherwise have appeared. Due to this study being conducted on a population of exclusively Veterans, these findings may not extrapolate to the general population, and since VA enrollment was not a requirement, these results cannot be applied to the VA healthcare system alone. It is also important to note that since no heterosexual or bisexual women and only one heterosexual man in our sample reported having taken PrEP, conclusions about the association of alcohol use with a history of PrEP use are limited in those subgroups. Due to small sample sizes, particularly in subgroups of people with unhealthy alcohol use, our analyses were unadjusted. Thus, residual confounding is likely, particularly by other factors (e.g., race, age, education) that may impact PrEP literacy.

## Conclusions

This study is the first to our knowledge to explore whether patterns of unhealthy alcohol use are associated with PrEP awareness, willingness, uptake, and healthcare discussions in a population-based sample of Veterans and across sexual and gender groups. Our findings were consistent with previous findings that PrEP awareness and uptake are extremely low and vary across sexual and gender groups and underlined that some important groups (e.g., women) are not receiving PrEP information or treatment. The impact of unhealthy alcohol use patterns on PrEP awareness and uptake was often minimal and varied across sexual and gender groups. However, unhealthy alcohol use patterns were associated with increased likelihood of having conversations with a doctor about PrEP. While this is encouraging due to the increased risk of HIV associated with alcohol use, the low rates of these conversations reported is concerning. Findings from the study suggest that future efforts are needed to increase PrEP awareness and uptake and to expand awareness of PrEP to groups historically less impacted by HIV; the idea that only sexual and gender minoritized groups are at risk is not consistent with current HIV epidemiology. Heterosexual women with patterns of unhealthy alcohol use may be an important target group for intervention research, and implementation research is needed to address gaps in PrEP care for all patients. These future efforts may be bolstered by the relatively higher rates of PrEP willingness observed in the present study, regardless of patterns of unhealthy alcohol use. Interventions targeting both unhealthy alcohol use and HIV prevention should capitalize on this willingness.

## Data Availability

Data are not publicly available due to institutional rules regarding data sharing.

## Abbreviations

AUD: Alcohol use disorder
AUDIT-C: Alcohol use disorder identification test–consumption
LGBTQ: Lesbian, gay, bisexual, transgender, queer
MSM: Men who have sex with men
PrEP: Pre–exposure prophylaxis
